# Combining point counts and autonomous recording units improves avian survey efficacy across elevational gradients on two continents

**DOI:** 10.1002/ece3.7678

**Published:** 2021-06-01

**Authors:** Anna Drake, Devin R. de Zwaan, Tomás A. Altamirano, Scott Wilson, Kristina Hick, Camila Bravo, José Tomás Ibarra, Kathy Martin

**Affiliations:** ^1^ Department of Forest and Conservation Sciences University of British Columbia Vancouver BC Canada; ^2^ ECOS (Ecosystem‐Complexity‐Society) Co‐Laboratory Center for Local Development (CEDEL) & Center for Intercultural and Indigenous Research (CIIR) Pontificia Universidad Católica de Chile Santiago Chile; ^3^ Wildlife Research Division National Wildlife Research Centre Environment and Climate Change Canada Ottawa ON Canada; ^4^ Pacific Wildlife Research Centre Environment and Climate Change Canada Delta BC Canada; ^5^ Institute of Ecology and Biodiversity Department of Ecological Sciences Faculty of Science Universidad de Chile Santiago Chile; ^6^ Millennium Nucleus Center for the Socioeconomic Impact of Environmental Policies (CESIEP) & Center of Applied Ecology and Sustainability (CAPES) Pontificia Universidad Católica de Chile Santiago Chile

**Keywords:** alpine, ARUs, avian diversity, biodiversity monitoring, bird surveys, high mountain biodiversity, species richness, subalpine

## Abstract

1. Accurate biodiversity and population monitoring is a requirement for effective conservation decision making. Survey method bias is therefore a concern, particularly when research programs face logistical and cost limitations.
2. We employed point counts (PCs) and autonomous recording units (ARUs) to survey avian biodiversity within comparable, high elevation, temperate mountain habitats at opposite ends of the Americas: nine mountains in British Columbia (BC), Canada, and 10 in southern Chile. We compared detected species richness against multiyear species inventories and examined method‐specific detection probability by family. By incorporating time costs, we assessed the performance and efficiency of single versus combined methods.
3. Species accumulation curves indicate ARUs can capture ~93% of species present in BC but only ~58% in Chile, despite Chilean mountain communities being less diverse. The avian community, rather than landscape composition, appears to drive this dramatic difference. Chilean communities contain less‐vocal species, which ARUs missed. Further, 6/13 families in BC were better detected by ARUs, while 11/11 families in Chile were better detected by PCs. Where survey conditions differentially impacted method performance, PCs mostly varied over the morning and with canopy cover in BC, while ARUs mostly varied seasonally in Chile. Within a single year of monitoring, neither method alone was predicted to capture the full avian community, with the exception of ARUs in the alpine and subalpine of BC. PCs contributed little to detected diversity in BC, but including this method resulted in negligible increases in total time costs. Combining PCs with ARUs in Chile significantly increased species detections, again, for little cost.
4. Combined methods were among the most efficient and accurate approaches to capturing diversity. We recommend conducting point counts, while ARUs are being deployed and retrieved in order to capture additional diversity with minimal additional effort and to flag methodological biases using a comparative framework.

## Graphical abstract

Using avian surveys conducted in comparable, high elevation, temperate mountain habitats at opposite ends of the Americas (British Columbia, Canada, and southern Chile), we show combined ARU and point count methods are among the most efficient and accurate approaches to capturing diversity. ARUs were predicted to capture ~93% of species present in British Columbia but only ~58% in Chile, despite Chilean mountain communities being less diverse. The use of combined methods enable studies to flag methodological biases using a comparative framework.

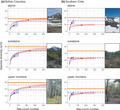

## 1 INTRODUCTION

Species surveys are used to determine the presence, relative abundance, and diversity of taxa over space and time (Roberts, 2011; Sauer et al., 2017; Schramm et al., 2020). As a cornerstone of many ecological studies, these metrics are used to identify biodiversity hotspots, infer the impact of natural or anthropogenic disturbances on communities, assess the effectiveness of management practices, and identify important habitats for species of conservation concern (e.g., Dorji et al., 2019; Friedlander et al., 2019; Ibarra & Martin, 2015; Rosenberg et al., 2017). For effective conservation decision making to occur, biases associated with any given survey technique should be quantified and, where possible, corrected for. When abundance and diversity data are compared across broad regions and divergent communities, any interaction between detection bias due to survey method and the landscapes and/or communities being surveyed is a concern. The use of multiple survey methods can highlight these problems and may improve project coverage and efficiency.

For terrestrial birds, point counts (PCs) have been the standard survey method for more than 80 years (Ralph et al., 1995). Point counts employ 1–2 trained observers to identify and count birds by sight and sound from a single location for a set period of time. Within the past 20 years, the use of autonomous recording units (ARUs) as an alternative to point count surveys has become increasingly popular (Darras et al., 2019). ARUs are installed at survey sites and record ambient sound that is then analyzed in the laboratory, with species identified by their vocalizations either manually or using automated identification software. Both methods have benefits and limitations as techniques for surveying avian diversity. Key among the benefits of point counts is the ability to visually identify species (Acevedo & Villanueva‐Rivera, 2006; Hutto & Stutzman, 2009; Vold et al., 2017) and use distance to obtain more accurate density estimates than can be assessed by audio alone (Shonfield & Bayne, 2017). Because point count observers can assess call direction and track individual birds, they outperform ARUs when calls occur outside the ARU microphone(s) “line‐of‐sight” (Castro et al., 2019). ARUs, on the other hand, overcome logistical constraints experienced by point counts that can impact species detections. ARUs can collect data simultaneously from multiple sites, allowing projects to survey during peak diel activity for both diurnal and nocturnal species (Goyette et al., 2011), and eliminating potential temporal bias present in point counts along lengthy transects (Darras et al., 2019). ARUs can be left in remote locations, such as high latitude and high elevation habitats, year‐round, and be programmed to start recording in spring before observers can safely access these regions (e.g., Shonfield & Bayne, 2017). ARUs can therefore better‐sample peak seasonal activity for resident species and detect shifts in bird phenology (Klingbeil & Willig, 2015). As inanimate objects, ARUs are also less likely to alter bird behavior compared to observers (Shonfield & Bayne, 2017, Darras et al., 2019, but see Hutto & Hutto, 2020). Finally, recordings provide a permanent record, allowing researchers to replay calls and seek assistance with difficult species identification, thereby reducing observer bias (Shonfield & Bayne, 2017).

In a meta‐analysis of the two methodologies, Darras et al. (2019) demonstrated that, on average, point counts and ARUs do not differ significantly in the diversity of species they detect. However, among studies, there are differences in performance by method that likely relate to the habitat and terrain surveyed (Castro et al., 2019; Celis‐Murillo et al., 2012; Klingbeil & Willig, 2015; Kułaga & Budka, 2019), and/or the behavior, vocalization characteristics, and rarity of the species monitored (Castro et al., 2019; Celis‐Murillo et al., 2009; Hutto & Stutzman, 2009). Even when diversity is comparable, methods may not be equivalent because they sample different subsets of the focal community (Venier et al., 2012). Thus, researchers should be cautious in assuming that ARUs and point counts are interchangeable in every system (Alquezar & Machado, 2015).

Effort is a consideration for research programs and point counts, and ARUs differ in their time costs. A point count is completed in a single site visit, while deploying an ARU and retrieving data entails a minimum of two site visits. However, ARU recordings can subsequently be intensively sampled without increased field costs or increased site disturbance. ARUs can have notable drawbacks in terms of processing time in the laboratory: without automated data processing, the time costs of uploading and interpreting audio files, replaying sections of audio, and then transcribing observations are greater than for detections and transcriptions of equivalent length point counts (e.g., this study; Alquezar & Machado, 2015; Celis‐Murillo et al., 2009). Even with automated processing, the need to manually validate detections can eliminate any time advantages over manual scanning (Joshi et al., 2017 but see Knight et al., 2020).

Given their field advantages, ARUs offer a compelling alternative to point counts at high elevation sites. Mountain habitats present challenging conditions in which to conduct avian surveys and, despite mountains supporting important bird diversity, most high elevation systems in the Americas are poorly monitored (Boyle & Martin, 2015). Point counts within these systems are limited by access (difficult terrain, late snowmelt, or poor infrastructure), and surveys are often disrupted by inclement weather. By necessity, mountain surveys are typically conducted in a linear fashion, upslope, or downslope, producing a temporal bias in point counts stratified by elevation. ARUs sidestep many of these challenges, yet few studies have compared the two methods in these environments.

In this study, we examined the performance of ARUs and point count surveys in detecting and quantifying avian diversity across a gradient of temperate mountain habitats in both North (Canada) and South America (Chile). In both Canada and Chile, sampling encompassed three structurally similar habitats across increasing elevations: densely forested upper montane, semi‐open subalpine, and highly exposed alpine. Using species detections at shared sites, we directly compared diversity index values and species accumulation curves produced by these two methods. We investigated the underlying causes of differences in diversity values obtained by each method by modeling detection probabilities of bird families by survey method within the two regions. In order to make recommendations for future monitoring protocols, we used a cost‐benefit analysis (i.e., time cost versus species richness return) to examine the efficiency of point counts and ARU sampling in isolation and for combined‐method protocols.

## 2 MATERIALS & METHODS

### 2.1 Study locations

**FIGURE 1:**
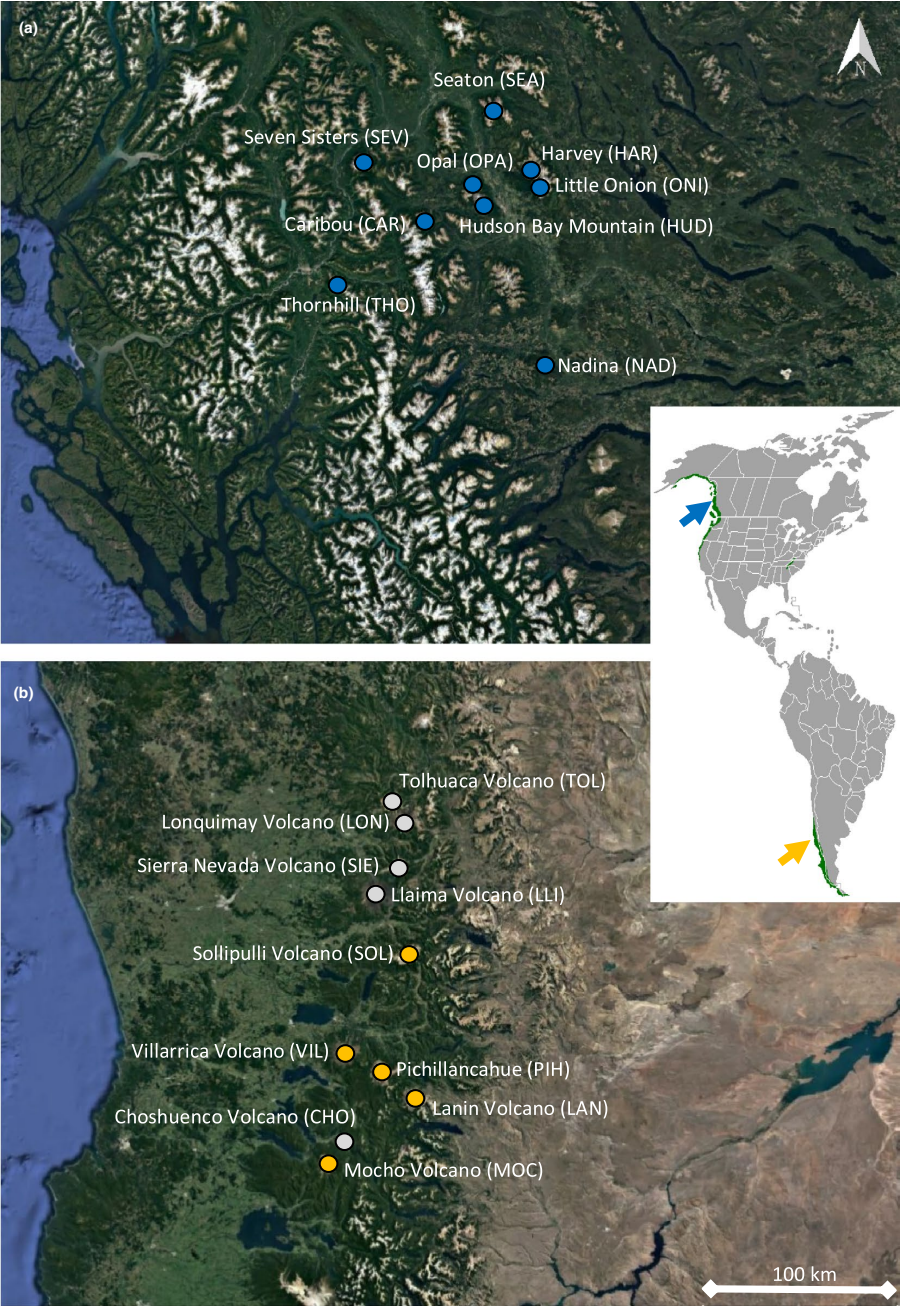
Location of the temperate mountains surveyed in British Columbia, Canada, and (54.325°N, 126.801°W) southern Chile (38.767°S, 70.704°W). Mountains in Chile where ARUs were not deployed are marked in gray. Images courtesy of Google Earth (13 December 2015). Arrows on the inset maps indicate the general region of the study sites in Canada (Blue) and Chile (Yellow)

In Canada (2019), we surveyed nine mountains in the D'ze Kant (Bulkley)‐Nechako and Kitimat‐Stikine regions of British Columbia (BC; 1,000–1,801 m elevation; Figure 1). In Chile (2018), we surveyed 10 mountains in La Araucanía and Los Ríos regions (1,000–1,700 m elevation; Figure 1). These mountains fall within the traditional unceded lands of the Wet'suwet'en, Gitxsan, and Tsimshian First Nations in BC and the Mapuche people in Chile. The farthest latitudinal and longitudinal distance among survey locations was 117 and 106 km, respectively, in BC, and 178 and 60 km, respectively, in Chile. Surveyed habitats across elevation gradients in both regions were classified as: montane habitat (≥50% tree cover, 1,000–1,557 m a.s.l.); subalpine (≥5%–50% tree cover, 1,169–1,658 m a.s.l.); and alpine (0%–5% tree cover, 1,319–1,801 m a.s.l; Boyle & Martin, 2015).

BC survey sites fall within five biogeoclimatic zones: Coastal Mountain Hemlock, Mountain Hemlock, Engelmann Spruce‐Subalpine Fir, Boreal Altai Fescue Alpine, and Coastal Mountain‐heather Alpine (British Columbia Ministry of Forests, Lands, Natural Resource Operations, & Rural Development, 2018). Montane habitat is primarily old growth conifer forest interspersed by avalanche chutes, producing age heterogeneity. The subalpine consists of woody shrubs, grasses, and perennial herbs with some tree cover, while the alpine is characterized by the presence of fescue grasses, heather, mosses, and lichens.

In Chile, montane habitats are dominated by old growth mixed broadleaf‐conifer forests, with about 10% midsuccessional forest. Subalpine habitat is a mix of highland herbaceous meadows, shrubs, and sparse patches of trees and/or krummholz. Perennial herbaceous plants, shrubs, few or no trees, and bare rock/scree characterize alpine habitat. Vegetation structure varies within and among mountains based on natural disturbances (i.e., volcanic eruptions) and/or land‐use history (Caviedes & Ibarra, 2017).

### 2.2 Point counts

Starting at sunrise, 95% of surveys were conducted within 5 hr to encompass peak bird activity. The remaining 5% of surveys occurred 5–6 hr after sunrise within subalpine and alpine habitats due to logistical constraints (total range: 04:53–11:24 hr in BC and 05:51–12:18 hr in Chile). Each mountain was surveyed from bottom to top (upslope) along transects with five designated point counts, 200 m apart, within each of the three habitat types for a total of 15 point counts per mountain. In BC, steep topography meant that the subalpine on Thornhill Mountain fits only four point count locations and that the alpine on Nadina Mountain was inaccessible until July. Thus, BC had 129 point count sites: one fewer subalpine site and five fewer alpine sites in total.

**FIGURE 2:**
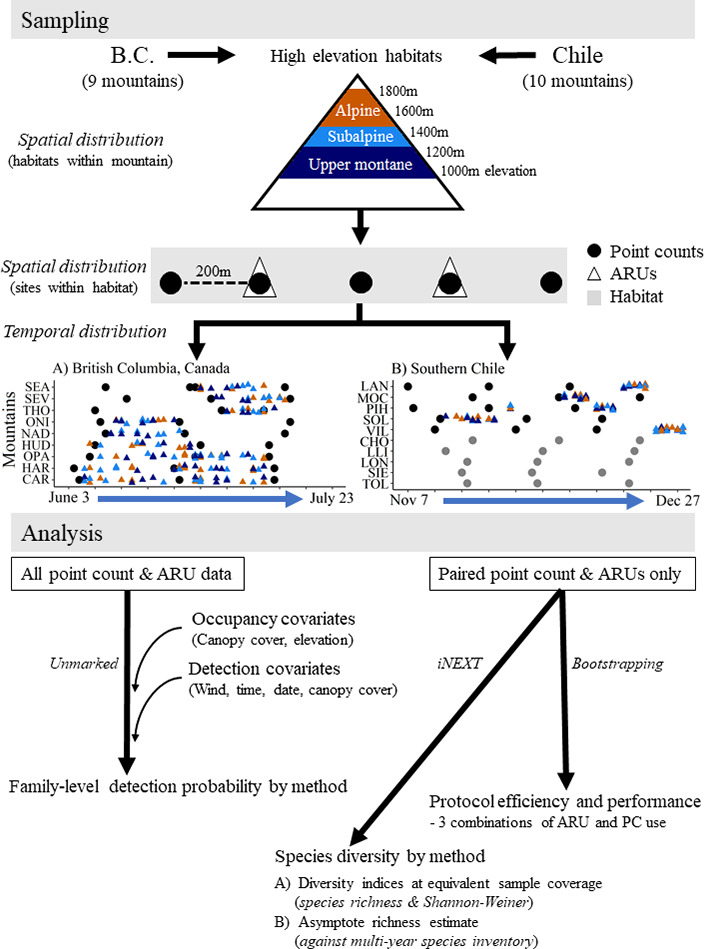
Study methodology. Surveys occurred during the breeding season of each region, 15 point count sites per mountain, five in each of three habitat types. Point counts were conducted on all mountains during early, mid, and late breeding season (3 rounds). ARUs were deployed on 9/9 mountains in British Columbia (BC), Canada, and on 5/10 mountains in southern Chile. ARUs were placed at 2 of the 5 point count sites within each habitat type. Detection models used data from all point count and ARU samples. Species richness models comparing method performance and protocol efficiency used only paired point count/ARU sites. Analysis methods are indicated beside the black arrows

During each 6‐min point count, birds were counted by sight and sound. Observers kept track of individual birds to minimize duplicate detections among point counts. Infinite radius detections were used to provide a fair comparison to ARU sampling; 95% of individuals in British Columbia and 99% of individuals in Chile were detected within 100 m. Point counts that occurred near habitat transition zones did not record species that called >100 m away if they were clearly within adjacent habitats or if they were in that direction and were unlikely to be in the focal habitat, based on their ecology. Counts were repeated three times within each respective breeding season: between May 30 and July 16 in BC, and between November 7 and December 21 in Chile, to assess detection probability and address seasonal variation in detection. Repeated site visits were separated by ~2 weeks (Figure 2).

### 2.3 Acoustic recordings and analysis

Song Meter SM4 Autonomous Recording Units (ARUs; Wildlife Acoustics Inc.^©^) with two omni‐directional microphones were deployed at two point count sites within each habitat and away from habitat transition zones (6 ARUs/mountain, >400 m apart) in both BC and Chile (Figure 2). In BC, 36 units were deployed on all nine mountains. As above, the alpine on Nadina Mountain was inaccessible resulting in ARUs being deployed at a total of 52 point count sites rather than 54. Units recorded for an average of 21 days within the BC breeding season: 10–20 days on six mountains, and 32–35 days on the remaining three mountains, between June 3 and July 15 (Figure 2). In Chile, six units were deployed on five mountains (30 point count sites in total) and recorded for an average of 6 days within the breeding season: 5–10 days each, between November 13 and December 28 (Figure 2). ARUs recorded at a sampling rate of 24,000 Hz in stereo wav format using default acoustic gain settings for the microphones. Units were mounted on a tree within several meters of the point count site, or on a PVC pipe at ~1.5 m height in the alpine. Units were programmed to record 30‐min on and 30‐min off, starting at sunrise and ending 5 hr after sunrise (5 × 30 min recordings/day). From the full deployment period, we randomly sampled two (BC) or three (Chile) different days per point count site. In BC, 4 days were selected for the three mountains where the ARUs were deployed for a longer period (early and late breeding season; Figure 2). To be comparable with point counts, we randomly chose one, 6‐min interval to analyze from each of the 5 × 30 min recordings on these days (5 × 6 min point counts/day). Thus, within each survey day, ARUs were sampled within hourly windows from between 0 and 1 hr after dawn (“hour 0”) to between 4 and 5 hr after dawn (“hour 4”). If any given hour(s) within the selected day had unfavorable conditions (wind or rain) that interfered with the audio, another day was selected randomly to obtain the missing time period(s). A total of 700 site‐surveys in BC and 450 site‐surveys in Chile were analyzed.

Sound recordings were analyzed using Audacity^®^ software (V2.3.0, Audacity Team, 2020). Three skilled observers reviewed all recordings: two in BC and one in Chile. All observers had experience conducting point counts in the same regions. In BC, both observers analyzed five of the same recordings to confirm detection consistency and conferred with each other on all recordings when species identification was uncertain. Spectrograms were scanned manually in stereo format as the observer listened to the recording. Species that were more difficult to identify were compared with recordings available on bioacoustics libraries such as the Xeno‐Canto Foundation (2019) and/or sent to other skilled ornithologists.

### 2.4 Abiotic variables

At each point count, we recorded average temperature and wind speed using a Kestrel 3500 weather meter (Nielsen‐Kellerman Company). We additionally scored wind as a categorical variable (Beaufort scale: 0–3) during point counts to allow for comparison with ARU wind scores that were assigned on the same scale based on interference with the audio recording. We also recorded percent canopy, understory (vegetation ~30 cm in height), shrub, and ground cover (tundra vegetation, snow, rock, and dead trees) within a 50 m radius of all point count sites. More canopy foliage in Chile was deciduous than in BC. Canopy cover value therefore increased with leaf‐out during the season in Chile, while values in BC were static.

### 2.5 Statistical analyses

#### 2.5.1 Total known species richness by habitat

We tallied the number of species known to be present within each habitat (total known species richness) using our most complete species list compiled between 2017 and 2019 at our field sites. This complete list included species identified at PCs, while walking transects between point count sites (K. Martin et al., unpublished data), as well as species identified in ARU recordings during this study. These values therefore represent the minimum total species richness for each community.

#### 2.5.2 Species diversity indices by survey method

**FIGURE 3:**
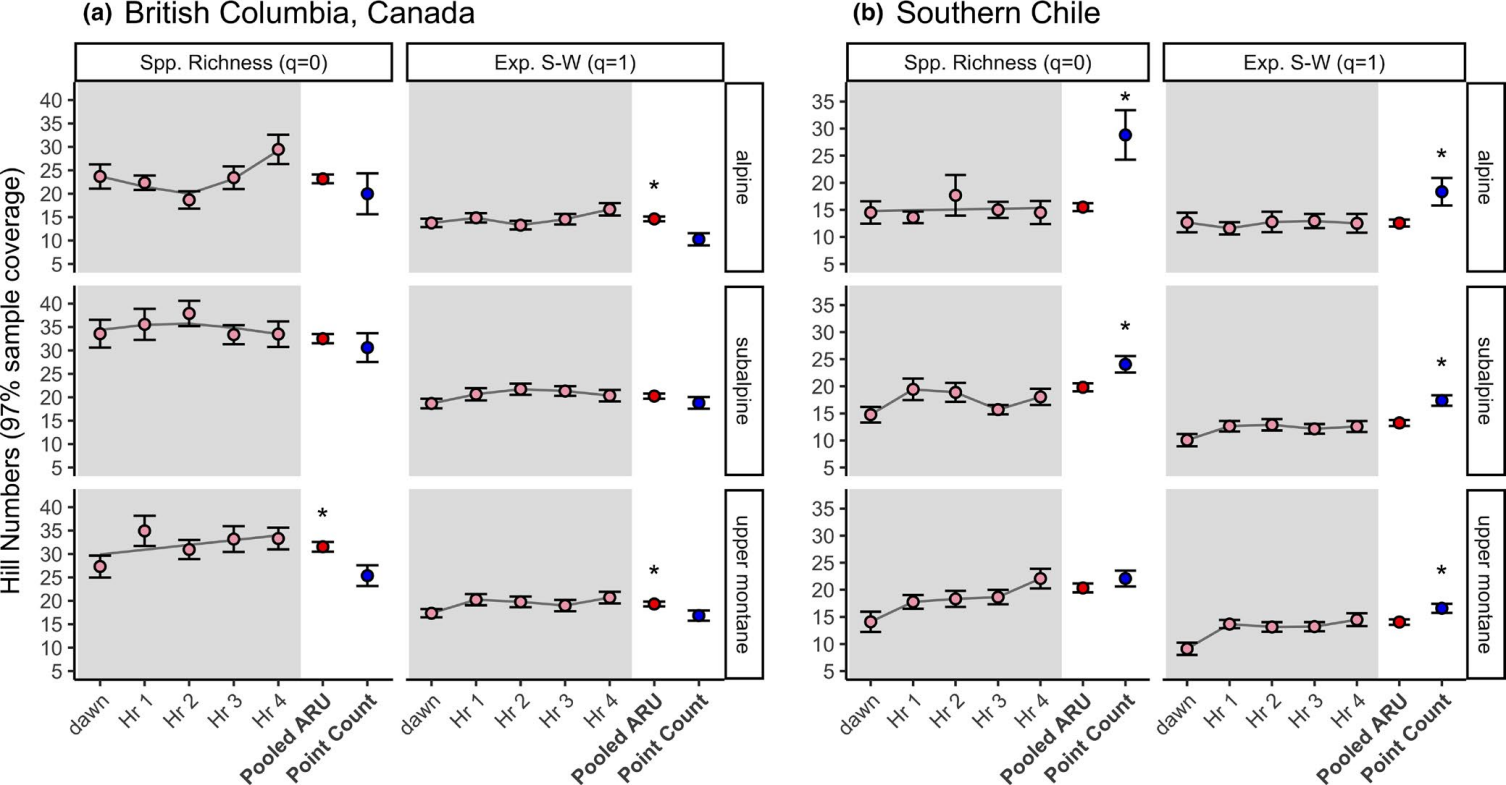
Species diversity values (±84% CI) obtained by PCs (blue filled circles) and ARUs (pink filled circles: five sampling times and red filled circles: pooled ARU sampling times) across three mountain habitats in British Columbia and southern Chile. Photographs of each habitat are given in Figure 4. Values presented are species richness (Hill number (*q*) = 0) and the effective number of species calculated by the exponential of the Shannon–Wiener Index (Hill number (*q*) = 1). All values are interpolated/extrapolated to 97% sample completeness. Values for both hourly ARU counts (from hour 0–hour 4 after dawn) and full morning ARU data, pooled, are presented. The gray line between hourly points is a spline fit to aid in visualizing potential temporal trends. Significant differences between methods are indicated by a *

All analyses were completed in program R (R Core Team, 2019). For diversity indices, we restricted our datasets to point count sites that were surveyed by both ARU and PC methods (BC: *n* = 52 sites, Chile: *n* = 30 sites). We then produced species accumulation curves for each method, using species incidence frequencies and the program iNEXT (Hsieh et al., 2016). For ARUs, within‐day hourly samples (hour 0 to hour 4) were modeled independently (BC: *n* = 44 (alpine) or 48 site‐surveys/habitat/hour; Chile: *n* = 30 site‐surveys/habitat/hour) and were also pooled over the whole morning (BC: *n* = 220 (alpine) or 240 site‐surveys/habitat; Chile: *n* = 150 site‐surveys/habitat) for comparison with PC survey data (BC: *n* = 48 (alpine) or 54 site‐surveys/ habitat; Chile: *n* = 30 site‐surveys/habitat). Sample sizes are larger for BC because we had access to more ARUs (see above). In both BC and Chile, diversity indices were calculated for each accumulation curve at 97% sample completeness through interpolation/extrapolation. This allowed for a fair comparison of the performance of each method, and each time‐period within ARU counts, regardless of sample size or effort. We report two diversity metrics (Hill numbers): (a) species richness (*q* = 0), and (b) the effective number of species calculated by the exponential of the Shannon–Wiener Index (*q* = 1), plus their 84% CI (MacGregor‐Fors & Payton, 2013) (Figure 3). Richness is presented as the count of species captured by either method. The exponential Shannon–Wiener value weights species by their frequency of occurrence and therefore minimizes the importance of species detected only once or twice by either method.

We used the “ChaoRichness“ function in iNext to predict the asymptote of the species richness accumulation curves of each method (Chao, 1984). This value is the predicted final species richness detected by each method if effort was increased. We compared these values to our minimum species diversity in each habitat.

#### 2.5.3 Detection probability by method

For species that were detected by one method only, we assessed the probability that this was due to a detection difference between methods versus chance using the Fisher's exact test on the frequency of detection by method across all site‐surveys (Fisher, 1992).

Because we were also interested in generalizable patterns of detection, we pooled species into family groups and assessed detection probability by method for each family using the R package “unmarked“ (Fiske & Chandler, 2011; Table A1). Detections at all point count sites were used for modeling detection probability, including sites that did not have ARUs installed (BC: *n* = 129 sites; Chile: *n* = 150 sites). The number of repeated surveys at each site ranged from 3 (PC‐only sites) to a maximum of 23 (five ARU surveys/day × 4 days and three PCs; BC: *n* = 1,087 site‐surveys; Chile: 900 site‐surveys). We only modeled families that occupied ≥15% of sites within any of the three habitat types. Modeling was then restricted to those habitats that encompassed 90% of the sites occupied by each family. For example, woodpecker detection was modeled for only upper montane forest (representing 94% of occupied sites) in British Columbia but for both upper montane (59% of occupied sites) and subalpine habitat (41% of occupied sites) in Chile (Table A1).

Because ARUs were sampled repeatedly within‐day with a spacing of ~1 hr (58 ± 13 min), we expected temporal autocorrelation between surveys within‐site and incorporated this into our models using a first‐order Markov covariate (Wright et al., 2016). We predicted that detection peaks might occur within‐season, over the morning, or over the range of canopy cover, and we therefore included quadratic terms for these variables.

Our baseline detection probability model was as follows:

detection ~ wind score + hours after sunrise + hours after sunrise^2^ + date + date^2^ + canopy cover + canopy cover^2^ + temporal autocorrelation term

And site occupancy probability was modeled as:

occupancy ~ site elevation + residuals of canopy cover by elevation.

Canopy cover residuals were used in the occupancy model to account for co‐linearity between elevation and canopy (i.e., trees become sparser at higher elevations). In Chile, canopy cover values at the time of sampling were used for modeling detection in order to account for leafing‐out, while maximum canopy cover at each site (reflective of habitat type) was used for modeling occupancy.

To our baseline detection model, we added an effect of method (ARU vs. PC) on detection plus interactions between method and three survey‐condition parameters where effects on detection were predicted to differ between ARU and PCs. These were as follows: canopy cover, hours after sunrise, and date. We tested the performance of the baseline model, the baseline + method model, and the seven possible models that included combinations of the three survey‐condition parameters. In total, nine detection models were tested for each bird family (Table A3).

We selected the best model for each family based on Quasi Akaike Information Criterion (QAIC), incorporating the over‐dispersion parameter ($\hat{c}$) for the most complex model (detection ~ baseline model + method + all three method interactions; Burnham & Anderson, 2002; MacKenzie et al., 2017; Mazerolle, 2017). Goodness‐of‐fit tests were run for these best models and, where $\hat{c}$ > 1, we inflate the CIs accordingly. We do not present output for any family where $\hat{c}$ > 4 (suggesting lack of fit; Mazerolle, 2017) or where $\hat{c}$ < 0.3 (indicating insufficient data). We report the 84% and 95% CIs: No overlap at the 84% CI is consistent with a significant difference (*p* < 0.05) between methods (Payton et al., 2003), while the 95% CI represents the 95% CI of the actual detection probability. Further detail on detection probability modeling is available in the Appendix A.

#### 2.5.4 Protocol efficiency and performance

We assessed the efficiency of single‐method and mixed‐method sampling protocols as the percent of the total community detected as a function of hours of effort. For ARUs, site visitation and sample processing costs were assessed at 40 min/site and 9 min/sample. For PCs, these values were 20 min/site and 7 min/sample. When protocols were mixed, we assumed that the visitation cost was shared for ARUs and PCs (i.e., that PCs were conducted when ARUs were deployed and/or retrieved). In protocols that involved three PCs per site, the additional PC incurred an additional visitation cost (20 min/site). We randomly sampled ARU and PC surveys with replacement (10,000 replicates) at each point count site to produce a bootstrapped mean species richness detected (±SE) across all sites for different sampling intensities of: ARUs alone (1–15 counts/site), PCs alone (1–3 counts/site), and PC plus ARU surveys (1 PC + 1–15 ARU counts/site, 2 PCs + 1–15 ARU counts/site, etc.). We identify the “best” protocols as those that detected the greatest percentage of the total community (i.e., our total known species richness) for the least effort.

## 3 RESULTS

### 3.1 Species diversity indices

In BC, at 97% predicted community coverage, PCs and pooled ARUs obtained equivalent species richness (*q* = 0) in both the alpine and the subalpine (Figure 3). Pooled ARUs obtained higher richness scores than PCs in the upper montane. When species were weighted by their frequency of occurrence in either dataset (*q* = 1), the methods performed equivalently in the subalpine, but pooled ARUs outperformed PCs in the alpine and upper montane (Figure 3). Thus, three of six comparisons in BC showed equivalent performance for the two methods and three indicated ARUs were superior, particularly in the upper montane.

**FIGURE 4:**
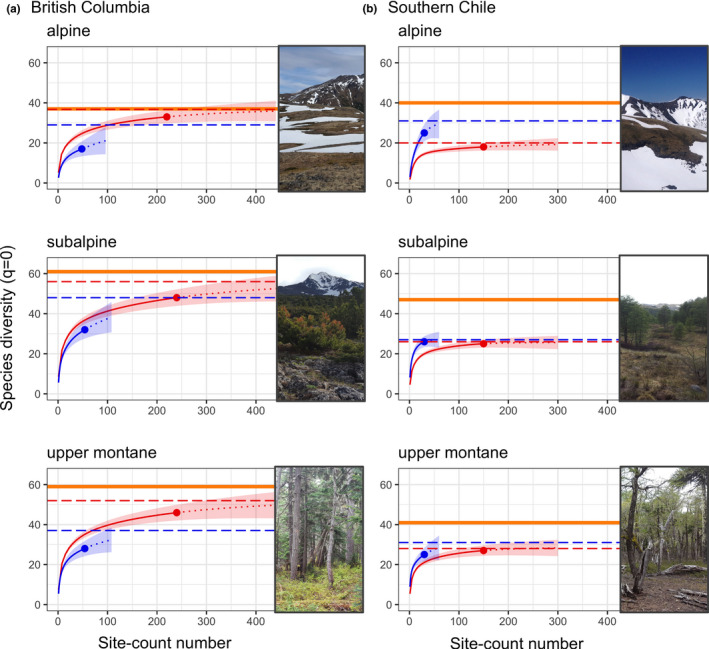
Species richness (*q* = 0) accumulation curves for PCs (blue) and ARUs (red) across three montane habitats in British Columbia and in southern Chile (± 95% CI). Dotted lines indicate the extrapolation of the species accumulation curve with increased effort while the dashed horizontal lines indicate the predicted final species richness obtained by each method (i.e., the predicted curve asymptote). The solid orange line indicates total known community richness based on multiyear habitat sampling and including all observations (see Methods)

In BC, ARU detections were more likely than point counts to intersect with the total known species richness of the alpine and subalpine (Figure 4). On average, pooled ARUs were predicted to capture 93% (range: 88%–100%) of the known community across all habitats, while PCs were predicted to capture 73% (63%–79%).

In Chile, at 97% predicted community coverage, PCs obtained greater species richness (*q* = 0) values than pooled ARUs in the subalpine and alpine (Figure 3). In upper montane forest, the richness obtained by both methods was equivalent. When first‐order diversity (*q* = 1) was assessed, PCs continued to be better than pooled ARUs at detecting species diversity, outperforming ARUs in the upper montane as well. Thus, five out of six comparisons in Chile indicated that PCs outperformed ARUs; the sixth showed a nonsignificant bias toward PCs (Figure 3).

For both methods in Chile, the predicted asymptotes of the species accumulation curves did not approach our total known species richness within each habitat (Figure 4). On average, pooled ARUs were predicted to capture 58% (range: 50%–68%) of the known community across all habitats, while PCs were predicted to capture 70% (57%–78%).

In both regions, dawn ARU counts detected lower or equivalent richness to ARU counts later in the morning (*q* = 0, Figure 3). The only exception was in the BC alpine, where dawn counts detected more species than counts two hours after dawn (*q* = 0, Figure 3). Although dawn recordings were often less rich, in Chile they detected two owl and one nightjar species that were not detected later in the morning (see Table A2 and below).

### 3.2 Species identified by only one method

In BC, ARUs detected all but one of the species recorded by point count observers plus an additional 17 species, or 29% of the diversity detected by both methods pooled (Table A2). Of these species, only Townsend's solitaire (*Myadestes townsendi*) was detected frequently enough by ARUs to indicate that the detection difference between methods was not due to chance (Fisher's exact test; *p* = 0.02; Table A2).

In Chile, 13 species, or 26% of the diversity captured by both methods pooled, were detected by point count observers but missed by ARUs (Table A2). Most of these were raptors (6/13) and ground‐tyrants (5/13; Tyrannidae). Of these 13 species, five were detected frequently enough by PCs to indicate that the detection difference between methods was not a product of chance (Fisher's exact test; *p* < 0.05; Table A2). These species were as follows: Bar‐winged cinclodes (*Cinclodes fuscus*), Dark‐faced ground‐tyrant (*Muscisaxicola maclovianus*), Spot‐billed ground‐tyrant (*M. maculirostris*), Ochre‐naped Ground‐tyrant (*M. flavinucha*), and Red‐backed hawk (*Geranoaetus polyosoma*). Four species, or 8% of the diversity captured by both methods pooled, were detected by ARUs but missed by PCs (Table A2). Three of these species were nocturnal and were detected only in dawn ARU recordings, the exception being the diurnal Austral pygmy owl (*Glaucidium nana*). None were detected frequently enough to exclude the possibility that the detection difference between methods was due to chance.

### 3.3 Family‐level detection probabilities by method

**FIGURE A1:**
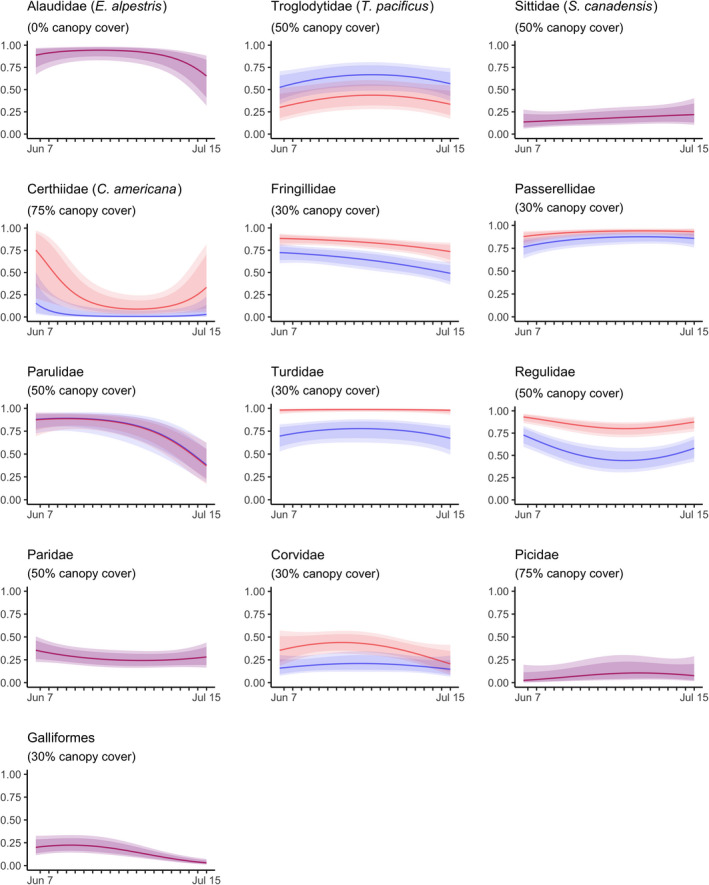
Detection probabilities for families in British Columbia (84 and 95% CI), by date (A1), hours after sunrise (A2), and canopy cover (A3), as predicted by the best model for each family based on QAIC and accounting for overdispersion ($\hat{c}$). ARU = red, PC = blue, no difference in method performance = purple. Dashed vertical lines in canopy cover plots indicate habitat transition points (alpine‐subalpine (5%), subalpine‐upper montane (50%)). Where applicable, values are plotted with canopy, date, and/or time held constant at: the midvalue for habitats modeled (as indicated), midseason, and midmorning

**FIGURE A2:**
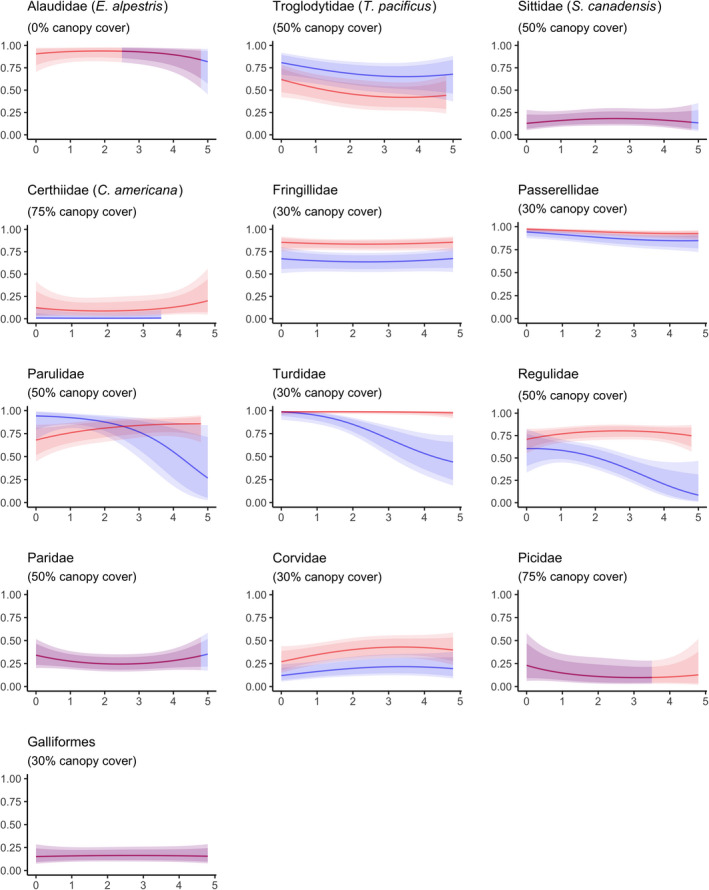
See Figure A1 caption

**FIGURE A3:**
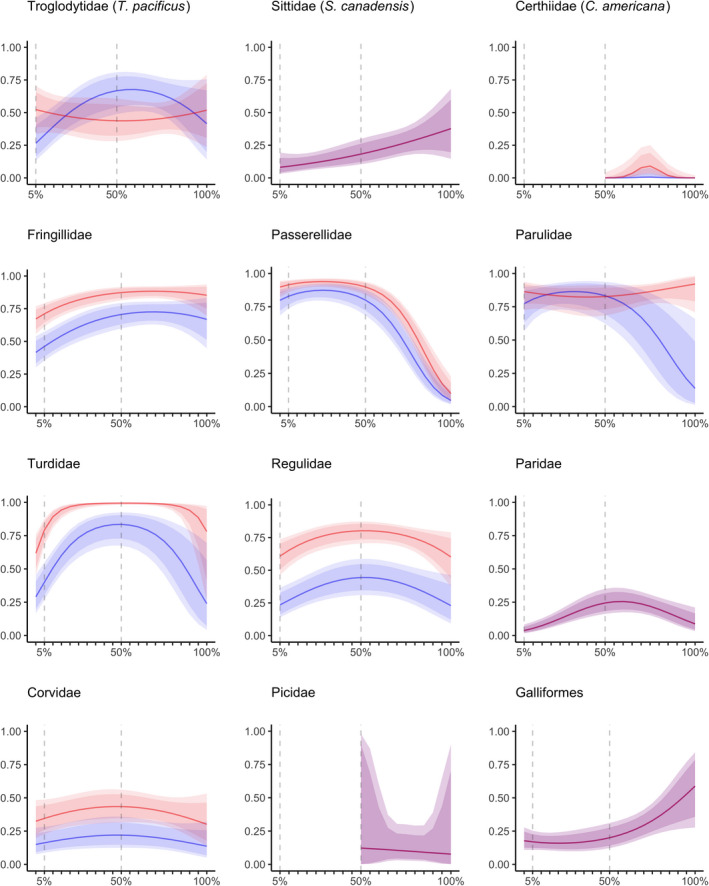
See Figure A1 caption

In BC, models supported an effect of methodology on detection for eight of the 13 families examined (Table A3). These were as follows: wrens (*Troglodytidae*), creepers (*Certhiidae*), finches (*Fringillidae*), sparrows (*Passerellidae*), warblers (*Parulidae*), thrushes (*Turdidae*), kinglets (*Regulidae*), and corvids (*Corvidae*). Six of these families were consistently better detected by ARUs, though for sparrows and creepers the advantage was minimal (Figures A1, A2, A3). Wrens were better detected by PCs within a narrow range of canopy cover (55%–75%); warblers showed a detection advantage for PCs in the early morning and for ARUs at sites with high canopy cover (Figures A1, A2, A3). Warblers, and thrushes, and kinglets showed an interaction between survey method and hours after sunrise: Detection probability declined over the morning for PCs but remained consistently high for ARUs (Figure A2). Wrens, warblers, and thrushes showed an interaction between method and canopy cover: Detection probability was more variable for PCs than for ARUs over the range of canopy cover (Figure A3). Finally, corvids showed an interaction between method and date, being better detected by ARUs midseason (Figure A1).

**FIGURE A4:**
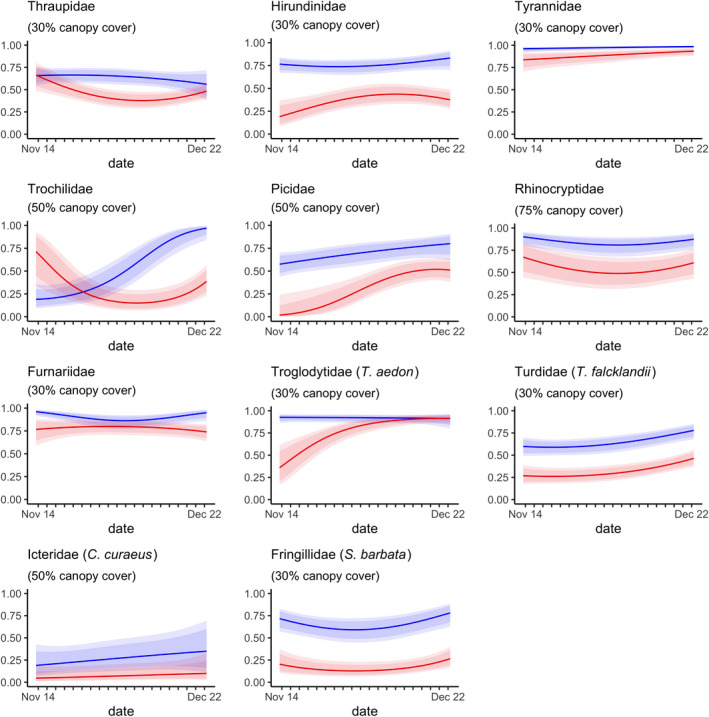
Detection probabilities for families in southern Chile (84 and 95% CI), by date (A4), hours after sunrise (A5), and canopy cover (A6) as predicted by the best model for each family based on QAIC and accounting for overdispersion ($\hat{c}$). ARU = red, PC = blue, method overlap = purple. Dashed vertical lines in canopy cover plots indicate habitat transition points (alpine‐subalpine (5%), subalpine‐upper montane (50%)). Where applicable, values are plotted with canopy, date, and/or time held constant at: the midvalue for habitats modeled (as indicated), midseason, and midmorning

**FIGURE A5:**
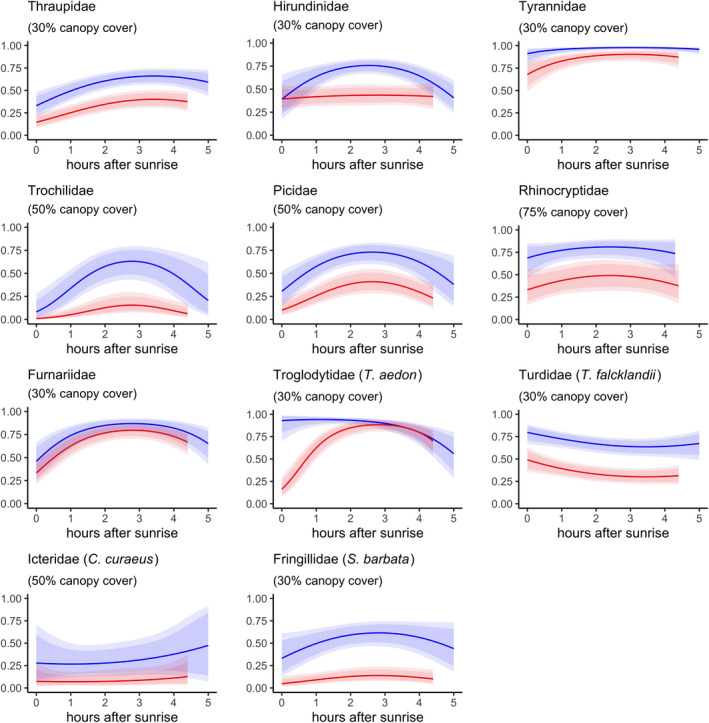
See Figure A4 caption

**FIGURE A6:**
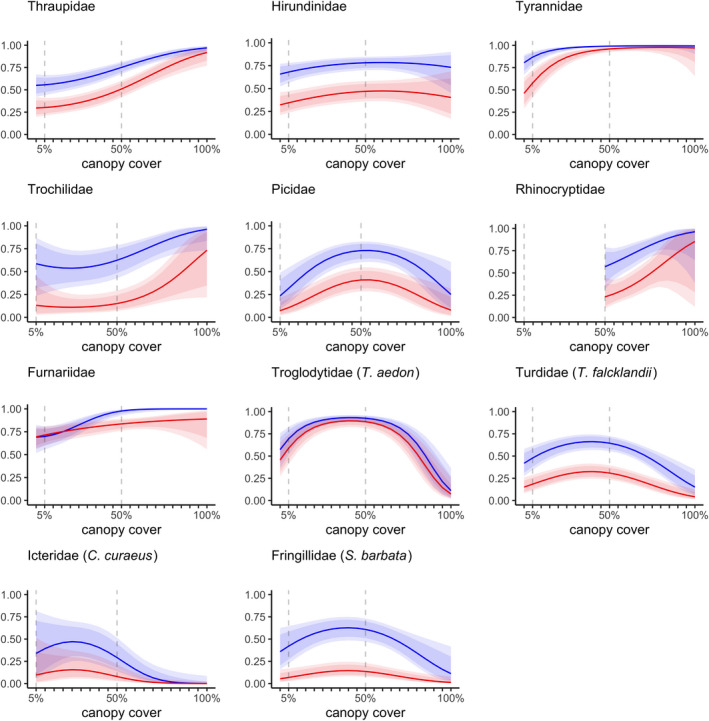
See Figure A4 caption

In Chile, detection models for all 11 families examined supported a methodology effect, with a higher detection probability for point counts than for ARUs (Table A3; Figures A4, A5, A6). Of these, six families showed an interaction between methodology and date. ARU detection probabilities for swallows (*Hirundinidae*), hummingbirds (*Trochilidae*), woodpeckers (*Picidae*), wrens (*Troglodytidae*), and tanager (*Thraupidae*) were either lower early in the monitoring period or exhibited a midseason dip. The detection probability of ovenbirds (*Furnariidae*) showed a midseason dip in point counts, but not ARUs (Figure A4). Ovenbirds additionally had lower detection probability with ARUs under conditions of high canopy cover. Swallows were better detected by point counts in the midmorning, while wrens were more poorly detected by ARUs in the early morning (Figure A5).

**FIGURE A7:**
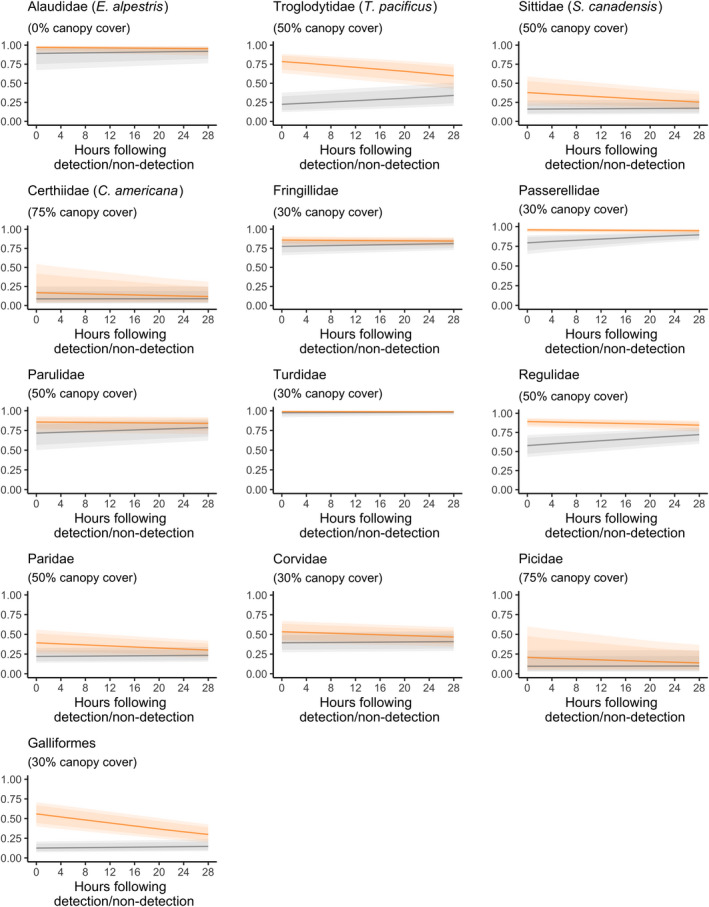
Predicted ARU detection probabilities (84 and 95% CI) for families in British Columbia (A7) and southern Chile (A8) as a function of time since a previous detection (orange) or nondetection (gray) at a given site. Note that our models constrained possible temporal autocorrelation to ≤48 hr since a detection/nondetection (i.e., values are forced to converge at 48 hr, see Methods). For predictions, canopy, date, and time are held constant at: the midvalue for habitats modeled (as indicated), midseason, and midmorning

**FIGURE A8:**
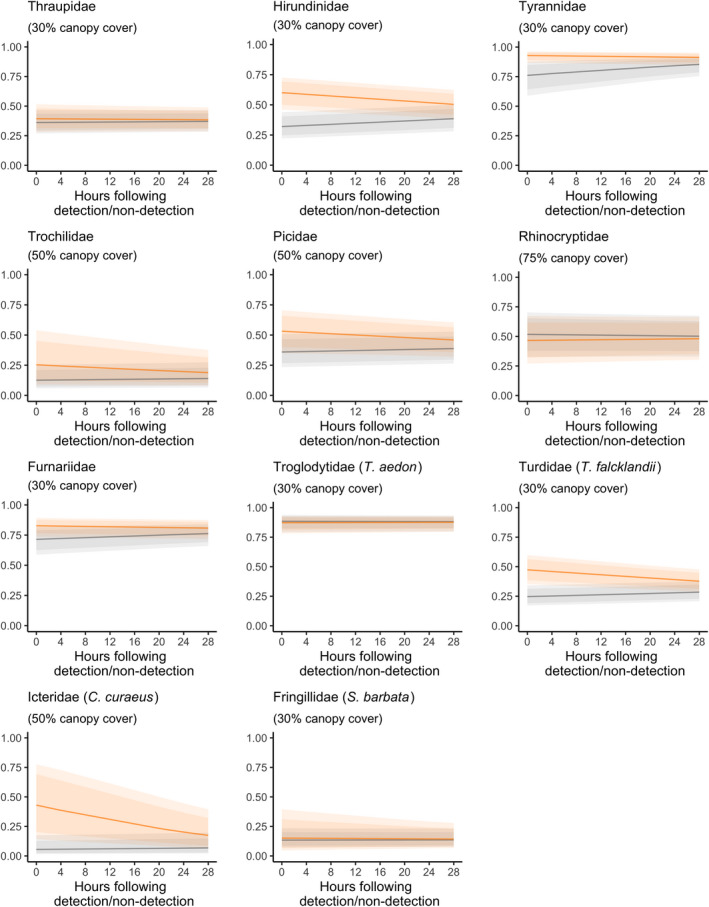
See Figure A7 caption

Temporal autocorrelation in ARU detection/nondetection significantly affected the detection probability of five families in British Columbia (*Trogloytidae*, *Sittidae*, *Passerellidae*, *Regulidae*, and *Galliformes*) and four families in Chile (*Hirundinidae*, *Tyrannidae*, *Turdidae*, and *Icteridae*; Figures A7 and A8).

### 3.4 Protocol efficiency and performance comparisons

**FIGURE 5:**
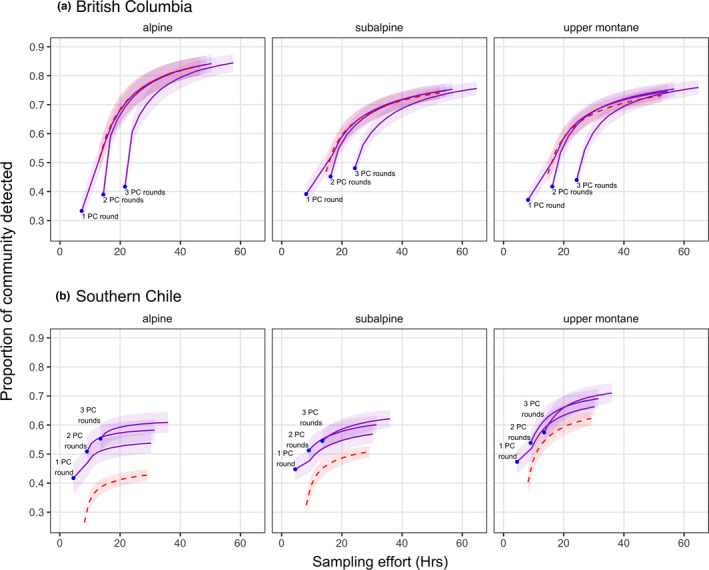
Efficiency of single‐method and dual‐method protocols as the bootstrapped proportion of the community (mean ± SE) detected with increasing monitoring hours across mountain habitats in British Columbia (BC) and southern Chile. Species detections were summed across all paired point count/ARU sites (BC: *n* = 16 (alpine) or 18 sites/habitat, Chile: *n* = 10 sites/habitat) for each level of effort. Point count returns (blue points) range in effort from 1 to 3 counts/site and are labeled. The ARU‐only protocol (red dashed curve) ranges in effort from 1 to 15 counts/site. Dual‐method protocols (purple curves) range from 1 to 15 ARU counts/site and vary in point count effort as labeled

In BC, species accumulation as a function of hours of effort was indistinguishable between ARU‐only protocols and one and two point count rounds plus ARU sampling (Figure 5a). This was because point counts did not contribute significantly to the total survey cost but, as shown above, they also did not contribute novel species to the accumulation curve. Three point count rounds and a mixed method that included point counts at this intensity were the least‐efficient sampling protocols in BC due to the increased cost associated with a third site visit. Surprisingly, in BC, a single ARU count/site detected more species than two point counts/site in the subalpine and more than three point counts/site in the alpine and upper montane, for equivalent or less effort (subalpine: 15 vs. 16 hr; alpine: 13 vs. 22 hr; upper montane: 15 vs. 24 hr; Figure 5a).

In Chile, ARUs alone were less efficient than point counts alone and less efficient than mixed methods due to fewer species detections. This was particularly notable in the Chilean alpine, where a single point count/site detected more species than 10 ARU counts/site and two point counts/site detected more species than were detected at our maximum ARU effort of 15 counts/site, for less effort (4.5 vs. 22 hr and 9 vs. 29 hr, respectively; Figure 5b). While species accumulation curves of mixed methods showed a large degree of overlap, a minimum of two point counts/site supplemented with ARUs appeared to be the best methodology for the subalpine and upper montane in Chile, and three point counts/site in the alpine boosted species detections enough to warrant the additional visitation cost (Figure 5b).

## 4 DISCUSSION

Avian surveys using ARUs can overcome major limitations experienced by point count methods. In our high elevation study system, these include site access limitations associated with remote, difficult terrain and late snowmelt as well as the disruption of surveys due to inclement weather. Detections of nocturnal species in dawn ARU recordings in this study also highlight the benefit of synchronous sampling across survey sites. Such advantages potentially make ARUs a powerful substitute for point counts in some challenging environments (e.g., Darras et al., 2019). Our results here, however, indicate that ARUs should be augmented by point counts: Dual methods allowed us to identify detection differences between methods where they were not anticipated. In our specific case, performance differences are likely attributable to differences in community composition between regions (as we discuss below). More generally however, our results show how dual methods enable monitoring programs to flag detection issues associated with individual survey methods and thus enhance comparisons across habitat types and ecosystems.

High mountain habitats in BC and Chile are structurally similar, yet ARU performance was markedly better in BC than in Chile. This illustrates that avian community composition can influence method performance as much as habitat composition. As in Klingbeil and Willig (2015), we believe differences in detection probability that favor point counts in Chile are largely due to visual detections of species. Raptor diversity is higher in Chile than BC and this largely silent group is best monitored by point counts. ARUs missed six raptor species that were detected by point counts (Table A2). Similarly, ground‐tyrants (*Tyrannidae*) rarely vocalize the following: The Xeno‐canto Foundation notes that, of all neotropical genera, ground‐tyrants and shrike‐tyrants are the most difficult to record. In our study, 5/9 tyrant species recorded by PCs were missed by ARUs (Table A2). Changes in vocalization frequency may also drive the seasonal variation in ARU detectability observed for 5/11 families in Chile. Song activity likely wanes when females are incubating or when pairs are feeding young (Moussus et al., 2009); yet, these individuals may remain visible during point counts when foraging. Interestingly, an interaction between method and seasonal detection probability was only seen in corvids in BC.

ARUs provide the ability to replay audio to confirm species identity for all vocalizations. In contrast, point counts are more vulnerable to observer effects: Individuals at point counts may miss species because they subconsciously screen out certain calls (“window species”; Kepler & Scott, 1981) and are overwhelmed with the number of calling species (Celis‐Murillo et al., 2009; Hutto & Stutzman, 2009), or because they mis‐identify infrequent calls (Bart, 1985; Celis‐Murillo et al., 2009). This may explain why ARUs perform so well in the species‐rich upper montane (Figure 3), and why a single ARU count/site in BC detected more species than a single point count/site, despite observation effort being equivalent (6 min/site; Figure 4a and Figure 5a). Two alternative explanations—that ARUs capture species’ peak activity because they sample a broader period of the morning, or that ARUs fail to screen out songs originating outside of their focal habitat and therefore overstate species diversity—were not well supported by our data. First, richness by hour showed no evidence of a peak in BC (Figure 3), nor was there an ARU detection peak over the morning within‐families (Figure A2). However, warblers, thrushes, and kinglets were all less likely to be detected by point counts later in the morning (Figure A2). Second, as vocalizations tend to carry upslope, we would expect ARUs near habitat transition zones to mis‐assign species to higher elevation habitats. Instead, ARUs in BC detected greater species diversity than point counts in upper montane habitat, not in the subalpine or alpine (Figure 3).

The ability to collect large amounts of data from ARUs is one of their advantages and, because the collection process itself is cheap, there is a temptation to obtain as much data as possible. However, the added time cost per sample associated with processing ARU data when compared to point count surveys needs to be carefully considered when planning monitoring protocols. Advances in automated processing may change this calculation (e.g., Knight et al., 2020), but additional time costs associated with training algorithms and proofing output will still apply (Joshi et al., 2017; Knight et al., 2017). Where ARUs perform poorly, as in the mountains of southern Chile, repeated sampling does not improve survey coverage (Figure 4). In other words, ARUs, like point counts, may miss large portions of communities regardless of effort. Monitoring programs should ascertain if this is the case before investing in increased ARU sampling.

In this study, greater ARU effort involved increased sampling within‐day: It is possible that sampling more days, with lower effort within‐day, would yield better returns. Temporal autocorrelation in detection probability supports this for a subset of families (Figures A7 and A8). These families appear to exhibit periodicity in vocalization or, possibly, proximity to the ARU and repeated sampling within day is less likely to provide novel information to surveys.

Our work expands on results from smaller scale studies that conclude dual methods are advantageous across a range of habitats (Celis‐Murillo et al., 2009, 2012 (in specific cases); Tegeler et al., 2012; Alquezar & Machado, 2015; Vold et al., 2017), as well as two broad‐scale studies within temperate and boreal forest (Holmes et al., 2014; Van Wilgenburg et al., 2017). Our novel comparison across structurally similar habitats in different geographic regions highlights the importance of avian community composition, in addition to habitat, in impacting method performance. We additionally show that the benefit‐to‐time‐cost ratio of dual methods that employ 1–2 point counts/site is comparable or better than single‐method approaches. Because our study system has relatively low species richness, our time costs for ARU transcription are relatively short. Where ARU processing is more time consuming, the benefits of employing dual methods should be more pronounced.

## 5 CONCLUSIONS

The inherent differences between point counts and ARUs mean their dual employment can identify strengths or weaknesses in the performance of either method across varied situations (e.g., habitats, temporal periods). When site visitation costs are shared, dual‐method surveys are efficient and can markedly increase community coverage. Where possible, we therefore recommend that point counts be conducted when ARUs are deployed and when their data are retrieved. As an additional benefit, with continually advancing ARU technology, data from dual methods will allow for standardization within long‐term monitoring projects and thus improved reliability of these valuable long‐term datasets. Additionally, if some ARU recordings and point counts are conducted in tandem, point count data can be used to assess site‐specific ARU detection radii (Van Wilgenburg et al., 2017; Yip et al., 2017). This would allow for better estimates of species densities from the audio data and help identify ARU species detection gaps (Vold et al., 2017). For occupancy studies, automated species detection software could then be trained and applied to longer sections of audio to efficiently search for species that have low ARU detection probabilities (Tegeler et al., 2012). Overall, we recommend the deployment of dual monitoring methods when conducting biodiversity assessments across larger spatial scales, diverse ecosystem types, or multiple geographic regions with differing wildlife community compositions.

## CONFLICT OF INTEREST

The authors declare that there is no conflict of interest.

## AUTHOR CONTRIBUTIONS

**Anna Drake:** Formal analysis (lead); Methodology (equal); Visualization (lead); Writing‐original draft (lead). **Devin R. de Zwaan:** Conceptualization (equal); Investigation (equal); Methodology (equal); Writing‐review & editing (equal). **Tomás A. Altamirano:** Conceptualization (equal); Investigation (equal); Methodology (equal); Writing‐review & editing (equal). **Scott Wilson:** Conceptualization (equal); Formal analysis (supporting); Investigation (equal); Methodology (equal); Writing‐review & editing (equal). **Kristina Hick:** Conceptualization (equal); Data curation (equal); Investigation (equal); Methodology (equal); Writing‐review & editing (equal). **Camila Bravo:** Data curation (equal); Investigation (equal); Methodology (equal); Writing‐review & editing (equal). **José Tomás Ibarra:** Conceptualization (equal); Methodology (equal); Writing‐review & editing (equal). **Kathy Martin:** Conceptualization (lead); Investigation (equal); Methodology (equal); Project administration (lead); Writing‐review & editing (equal).

## Data Availability

The datasets produced and analyzed in this study are available via Dryad [https://datadryad.org; data DOI: https://doi.org/10.5061/dryad.s4mw6m96d].

## APPENDIX A

### Method details for detection probability

#### Restriction of habitats modeled

We restricted analyses for each family to the habitats that encompassed 90% of the sites occupied by this family (Table A1). Where modeling was restricted to one or two habitats, time‐of‐day effects for point count detections were restricted to the window of time in which these habitats were surveyed. Similarly, we did not model ARU detection probability beyond the maximum time randomly sampled in a given habitat. For example, ARU sampling in Chile did not extend beyond 4.5 hr after sunrise and we therefore do not model predicted ARU detection probabilities after this time (Figure A5).

#### Incorporating temporal autocorrelation

Because ARUs were repeatedly sampled within‐day with a spacing of ~1 hr (58 ± 13 min), we expected temporal autocorrelation between surveys within‐site and incorporated it into our models using a first‐order Markov covariate (Wright et al., 2016). To do this, elapsed hours between each repeated survey (both ARU and PC) within‐site were calculated. This value was then scaled to produce a temporal proximity value that approached 1 as repeated surveys approached each other in time and became 0 when surveys were ≥48 hr apart. All first observations at a given site were assigned a temporal autocorrelation value equal to the family‐specific mean probability of detection within the dataset (*P* = number of detections/total number of surveys). The value of the temporal autocorrelation term for subsequent surveys was calculated as:

(1 − *P*)* temporal proximity + *P*, when a detection occurred in the previous survey
(0 − *P*)* temporal proximity + *P*, when a detection did not occur in the previous survey

Values of the temporal autocorrelation term therefore ranged from 0 to 1 and approached the mean probability of detection (i.e., the metric became uninformative) as survey spacing approached 48 hr.

#### Covariates selection in unmarked

Temperature at point count sites was not included independently in our detection models as it was correlated with hours after sunrise and/or with date (BC: Pearson's *r* = 0.20 and 0.42, respectively; Chile: *r* = 0.34, 0.09). We did not include other habitat covariates because these were moderately to strongly correlated with canopy (BC: Pearson's |*r*| = 0.52–0.88; Chile: |*r*| = 0.38–0.86) except for shrub cover (BC: |*r*| = 0.05; Chile: |*r*| = 0.27).

#### Determination of model fit and $\hat{c}$

We ran Mackenzie and Bailey goodness‐of‐fit (GOF) tests (1,000 iterations) to assess model fit and estimate $\hat{c}$. For all GOF tests, we restricted the number of repeated surveys per site to 10: the three PCs plus a random sample of seven ARU counts per site. This circumvented a known problem with the Mackenzie and Bailey GOF test, where extremely rare detection histories (which occur more frequently with greater repeated sampling within‐site) inflate the Pearson's chi‐square test statistic (MacKenzie et al., 2017).

### Method details for ARU versus PC efficiency

We assessed the efficiency of single‐method and mixed‐method sampling protocols as the percent of the total community detected as a function of hours of effort. For ARUs, protocol effort was calculated as the sum of (a) the average time required to visit each site to deploy the ARU (20 min/site), (b) the time required to revisit each site to collect data files (20 min/site) and (c) the time required to load, analyze, and transcribe detections for each 6‐min audio recording (9 min/sample). For PCs, effort was calculated as the sum of (a) the average time required to visit each site (20 min/site) and (b) the time required to conduct and transcribe detections for each 6 min count (7 min/sample).

**TABLE A1:** Species pooled to produce family‐level detection probabilities in (a) British Columbia and (b) Chile

Family	Species	Sites occupied			Detection analysis restricted to:
		Alpine (*n* = 40)	Subalpine (*n* = 44)	Upper montane (*n* = 45)	
(a) British Columbia, Canada					
Alaudidae	*Eremophila alpestris*	18	2	0	Alpine
Certhiidae	*Certhia Americana*	0	0	7	Upper montane
Corvidae	*Corvus corax* *C. brachyrhynchos* *Perisoreus canadensis* *Nucifraga columbiana* *Cyanocitta stelleri*	10	25	26	All habitats
Fringillidae	*Leucosticte tephrocotis* *Pinicola enucleator* *Loxia curvirostra* *L. leucoptera* *Spinus pinus* *Coccothraustes vespertinus*	35	42	44	All habitats
Galliformes	*Dendragapus obscurus* *D. fuliginosus* *Lagopus muta* *L. leucura* *L. lagopus* *Falcipennis canadensis*	25	19	10	All habitats
Paridae	*Poecile gambeli* *P. atricapillus* *P. hudsonicus* *P. rufescens*	0	16	34	Subalpine + upper montane
Parulidae	*Cardellina pusilla* *Leiothlypis peregrina* *Setophaga townsendi* *S. coronate* *S. striata* *Vermivora celata*	8	42	42	Subalpine + upper montane
Passerellidae	*Zonotrichia leucophrys* *Z. atricapilla* *Junco hyemalis* *Spizella passerina* *Melospiza lincolnii* *Passerella iliaca* *Passerculus sandwichensis* *Pooecetes gramineus*	39	44	38	All habitats
Picidae	*Hylatomus pileatus* *Colaptes auratus* *Picoides dorsalis* *Leuconotopicus villosus* *Sphyrapicus ruber*	0	1	15	Upper montane
Regulidae	*Regulus calendula* *R. satrapa*	5	38	42	Subalpine + upper montane
Sittidae	*Sitta canadensis*	0	12	29	Subalpine + upper montane
Troglodytidae	*Troglodytes pacificus*	3	22	33	Subalpine + upper montane
Turdidae	*Turdus migratorius* *Ixoreus naevius* *Catharus guttatus* *C. ustulatus* *Myadestes townsendi*	22	41	45	All habitats

Family	Species	Sites occupied			Detection analysis restricted to:
		Alpine (*n* = 50)	Subalpine (*n* = 50)	Upper montane (*n* = 50)	
(b) Southern Chile					
Fringillidae	*Spinus barbata*	11	47	43	All habitats
Furnariidae	*Geositta rufipennis* *Upucerthia saturatior* *Cinclodes fuscus* *C. oustaleti* *Aphrastura spinicauda* *Leptasthenura aegithaloides* *Sylviorthorhynchus desmursii* *Pygarrhichas albogularis* *Asthenes pyrrholeuca*	35	50	50	All habitats
Hirundinidae	*Tachycineta meyeni* *Pygochelidon cyanoleuca*	31	47	46	All habitats
Icteridae	*Curaeus curaeus*	4	26	12	Subalpine + upper montane
Picidae	*Dryobates lignarius* *Colaptes pitius* *Campephilus magellanicus*	0	30	43	Subalpine + upper montane
Rhinocryptidae	*Pteroptochos tarnii* *Scelorchilus rubecola* *Scytalopus magellanicus*	0	4	46	Upper montane
Thraupidae	*Phrygilus patagonicus* *P. unicolor* *Melanodera xanthogramma* *Diuca diuca*	31	48	49	All habitats
Trochilidae	*Sephanoides sephaniodes* *Oreotrochilus leucopleurus*	3	26	38	Subalpine + upper montane
Troglodytidae	*Troglodytes aedon*	11	48	40	All habitats
Turdidae	*Turdus falcklandii*	14	47	45	All habitats
Tyrannidae	*Elaenia albiceps* *Muscisaxicola maculirostris* *M. flavinucha* *M. maclovianus* *M. albilora* *Xolmis pyrope* *Colorhamphus parvirostris* *Agriornis montanus* *A. lividus*	42	50	50	All habitats

Note

Tables additionally present the number of sites occupied by each family out of the total number of sites surveyed (*n*) in each habitat and the habitats to which detection modeling was restricted (*see* Methods). Where one or two habitats encompassed 90% of the sites that were occupied by a given family, detection was modeled only for these habitats. Where families were more evenly distributed, all habitats were modeled.

**TABLE A2:** Species detection rate by method at paired survey sites in British Columbia, Canada (a) and Southern Chile (b)

Family	Species	English common name	Percentage of site‐surveys with detection	
			ARU (*n* = 700)	PC (*n* = 156)
(a) British Columbia, Canada				
Alaudidae	*Eremophila alpestris*	Horned Lark	16.4	7.7
Bombycillidae	*Bombycilla cedrorum*	Cedar Waxwing	0.4	0.0
Cardinalidae	*Piranga ludoviciana*	Western Tanager	0.0	0.6
Certhiidae	*Certhia americana*	Brown Creeper	2.6	1.3
Columbidae	*Patagioenas fasciata*	Band‐tailed Pigeon	0.1	0.0
Corvidae	*Nucifraga columbiana*	Clark's Nutcracker	7.4	1.9
	*Corvus corax*	Common Raven	1.7	0.0
	*Perisoreus canadensis*	Canada Jay	15.0	6.4
	*Cyanocitta stelleri*	Steller's Jay	0.6	0.0
Fringillidae	*Leucosticte tephrocotis*	Gray‐crowned Rosy‐Finch	4.1	0.0
	*Pinicola enucleator*	Pine Grosbeak	3.6	2.6
	*Spinus pinus*	Pine Siskin	70.1	50.0
	*Loxia curvirostra*	Red Crossbill	0.4	1.9
	*Loxia leucoptera*	White‐winged Crossbill	6.7	6.4
Galliformes	*Dendragapus obscurus*	Dusky Grouse	3.4	1.9
	*Lagopus muta*	Rock Ptarmigan	1.3	1.9
	*Dendragapus fuliginosus*	Sooty Grouse	4.4	3.8
	*Falcipennis canadensis*	Spruce Grouse	0.1	0.6
	*Lagopus lagopus*	Willow Ptarmigan	6.4	2.6
	*Lagopus leucura*	White‐tailed Ptarmigan	0.4	0.6
Motacillidae	*Anthus rubescens*	American Pipit	26.0	22.4
Paridae	*Poecile atricapillus*	Black‐capped Chickadee	0.9	0.0
	*Poecile hudsonicus*	Boreal Chickadee	1.3	5.1
	*Poecile rufescens*	Chestnut‐backed Chickadee	1.3	0.0
	*Poecile gambeli*	Mountain Chickadee	8.0	7.7
Parulidae	*Setophaga striata*	Blackpoll Warbler	2.0	1.9
	*Vermivora celata*	Orange‐crowned Warbler	3.3	0.6
	*Leiothlypis peregrina*	Tennessee warbler	0.1	0.0
	*Setophaga townsendi*	Townsend's Warbler	13.1	12.2
	*Cardellina pusilla*	Wilson's Warbler	22.7	19.9
	*Setophaga coronata*	Yellow‐rumped Warbler	42.1	38.5
Passerellidae	*Spizella passerina*	Chipping Sparrow	16.9	9.0
	*Junco hyemalis*	Dark‐eyed Junco	59.7	39.7
	*Passerella iliaca*	Fox Sparrow	36.0	16.0
	*Zonotrichia atricapilla*	Golden‐crowned Sparrow	40.9	24.4
	*Melospiza lincolnii*	Lincoln's Sparrow	9.9	3.8
	*Passerculus sandwichensis*	Savannah Sparrow	26.4	27.6
	*Pooecetes gramineus*	Vesper Sparrow	0.6	0.0
	*Zonotrichia leucophrys*	White‐crowned Sparrow	1.1	0.6
Picidae	*Picoides dorsalis*	American Three‐toed Woodpecker	2.1	0.6
	*Leuconotopicus villosus*	Hairy Woodpecker	0.3	0.0
	*Hylatomus pileatus*	Pileated Woodpecker	0.1	0.0
	*Sphyrapicus ruber*	Red‐breasted sapsucker	0.1	0.0
Regulidae	*Regulus satrapa*	Golden‐crowned Kinglet	25.9	17.9
Regulidae	*Regulus calendula*	Ruby‐crowned Kinglet	28.7	15.4
Scolopacidae	*Tringa solitaria*	Solitary Sandpiper	0.3	0.0
	*Gallinago delicata*	Wilson's Snipe	1.4	0.6
Sittidae	*Sitta canadensis*	Red‐breasted Nuthatch	9.4	7.7
Strigidae	*Glaucidium gnoma*	Northern Pygmy Owl	0.1	0.0
Trochilidae	*Selasphorus rufus*	Rufous Hummingbird	2.5	1.3
Troglodytidae	*Troglodytes pacificus*	Pacific Wren	31.3	26.3
Turdidae	*Turdus migratorius*	American Robin	31.0	13.5
	*Catharus guttatus*	Hermit Thrush	55.7	26.9
	*Catharus ustulatus*	Swainson's Thrush	6.1	4.5
	***Myadestes townsendi***	**Townsend's Solitaire**	**3.0**	**0.0**
	*Ixoreus naevius*	Varied Thrush	50.6	23.1
Tyrannidae	*Contopus cooperi*	Olive‐sided Flycatcher	1.0	0.0
	*Empidonax difficilis*	Pacific‐slope Flycatcher	1.9	0.0

Family	Species	English common name	Percentage of site‐surveys with detection	
			ARU (*n* = 450)	PC (*n* = 180)
(b) Southern Chile				
Anatidae	*Chloephaga poliocephala*	Ashy‐headed Goose	0.0	0.6
Caprimulgidae	*Caprimulgus longirostris*	Band‐Winged Nightjar	0.2	0.0
Cathartidae	*Vultur gryphus*	Andean Condor*	0.0	0.6
Columbidae	*Patagioenas araucana*	Chilean Pigeon	4.0	5.6
Falconidae	*Accipiter chilensis*	Chilean Hawk*	0.0	0.6
	*Buteo ventralis*	Rufous‐tailed Hawk	0.0	0.6
	*Caracara plancus*	Southern Crested Caracara	0.0	1.1
	*Falco sparverius*	American Kestrel	0.0	0.6
	***Geranoetus polyosoma***	**Red‐backed Hawk**	**0.0**	**4.4**
	*Milvago chimango*	Chimango Caracara	0.4	1.1
Fringillidae	*Spinus barbata*	Black‐chinned Siskin	8.9	48.9
Furnariidae	*Aphrastura spinicauda*	Thorn‐tailed Rayadito	36.2	53.3
	*Asthenes pyrrholeuca*	Sharp‐billed Canastero	0.7	0.6
	***Cinclodes fuscus***	**Bar‐winged Cinclodes**	**0.0**	**3.3**
	*Cinclodes oustaleti*	Gray‐flanked Cinclodes	8.2	14.4
	*Geositta rufipennis*	Rufous‐banded Miner	4.4	9.4
	*Leptasthenura aegithaloides*	Plain‐mantled Tit‐spinetail	4.0	11.7
	*Pygarrhichas albogularis*	White‐Throated Treerunner	12.7	22.2
	*Sylviorthorhynchus desmursii*	Des Murs`s Wire‐tail	0.2	1.7
	*Upucerthia saturatior*	Patagonian Forest Earthcreeper	9.6	7.2
Hirundinidae	*Pygochelidon cyanoleuca*	Blue‐and‐white Swallow	6.2	20.6
	*Tachycineta meyeni*	Chilean Swallow	24.0	46.1
Icteridae	*Curaeus curaeus*	Austral Black Bird	7.1	11.7
Passerellidae	*Zonotrichia capensis*	Rufous‐collared Sparrow	16.4	21.1
Picidae	*Campephilus magellanicus*	Magellanic Woodpecker	8.2	12.2
	*Colaptes pitius*	Chilean Flicker	2.9	6.7
	*Dryobates lignarius*	Striped Woodpecker	3.8	8.9
Psittacidae	*Enicognathus ferrugineus*	Austral Parakeet	4.7	7.2
Rhinocryptidae	*Pteroptochos tarnii*	Black‐throated Huet‐Huet	3.8	10.0
	*Scelorchilus rubecola*	Chucao Tapaculo	6.7	11.7
	*Scytalopus magellanicus*	Magellanic Tapaculo	3.6	10.0
Strigidae	*Bubo virginianus magellanicus*	Great Horned Owl (Magellanic)	0.4	0.0
	*Strix rufipes*	Rufous‐legged Owl	0.9	0.0
	*Glaucidium nana*	Austral Pygmy Owl	1.3	0.0
Thraupidae	*Melanodera xanthogramma*	Yellow‐Bridled finch	7.3	11.7
	*Phrygilus patagonicus*	Patagonian Sierra‐finch	34.7	50.6
	*Phrygilus unicolor*	Plumbeous Sierra‐finch	0.2	3.3
Threskiornithidae	*Theristicus melanopis*	Black‐faced Ibis	0.2	1.1
Trochilidae	*Sephanoides sephaniodes*	Green‐Backed Firecrown	11.8	15.0
Troglodytidae	*Troglodytes aedon*	Southern House Wren	40.7	62.8
Turdidae	*Turdus falcklandii*	Austral Thrush	26.9	39.4
Tyrannidae	*Agriornis lividus*	Great Shrike‐tyrant*	0.0	0.6
	*Agriornis montanus*	Black‐billed Shrike‐tyrant*	0.0	1.1
	*Colorhamphus parvirostris*	Patagonian Tyrant	0.2	0.6
	*Elaenia albiceps*	White‐crested Elaenia	60.2	68.3
	*Muscisaxucika albilora*	White‐browed Ground‐tyrant	1.6	20.6
	***Muscisaxicola flavinucha***	**Ochre‐naped Ground‐tyrant***	**0.0**	**3.9**
	***Muscisaxicola maclovianus***	**Dark‐faced Ground‐tyrant***	**0.0**	**4.4**
	***Muscisaxicola maculirostris***	**Spot‐billed Ground‐tyrant**	**0.0**	**1.7**
	*Xolmis pyrope*	Fire‐eyed Diucon	25.8	42.8

Note

Species in bold were entirely missed by one method but detected frequently enough by the other to indicate that this detection failure was not due to chance (Fisher exact test, *p* < 0.05). Starred species in Chile (*) were only visually identified, never heard, during point counts.

**TABLE A3:** Best performing detection models for each family by region based on QAIC and accounting for overdispersion ($\hat{c}$)

Model type	Detection ~	British Columbia	Southern Chile
**Baseline**	wind score + hours after sunrise + hours after sunrise^2^ + date + date^2^ + canopy cover + canopy cover^2^ + temporal autocorrelation term	Alaudidae Galliformes Paridae Picidae Sittidae	—
Method	**Baseline** + method	Certhiidae Fringillidae Passerellidae	Fringillidae Icteridae Rhinocryptidae Turdidae Tyrannidae
Method × Time	**Baseline** + method + method: hours after sunrise + method: hours after sunrise^2^	Regulidae	—
Method × Date	**Baseline** + method + method: date + method:date^2^	Corvidae	Picidae Thraupidae Trochilidae
Method × Canopy	**Baseline** + method + method: canopy + method: canopy^2^	Troglodytidae	
Method × Time + Method × Date	**Baseline** + method + method: hours after sunrise + method: hours after sunrise^2^ + method: date^2^ + method: date^2^	—	Hirundinidae Troglodytidae
Method × Time + Method × Canopy	**Baseline** + method + method: hours after sunrise + method: hours after sunrise^2^ + method: canopy + method: canopy^2^	Parulidae Turdidae	—
Method × Date + Method × Canopy	**Baseline** + method + method: date^2^ + method: date^2^ + method: canopy + method: canopy^2^	—	Furnariidae
Method × Time + Method × Date + Method × Canopy	**Baseline** + method + + method: hours after sunrise + method: hours after sunrise^2^ + method: date^2^ + method: date^2^ + method: canopy + method: canopy^2^	—	—

Note

The bolded "Baseline" term indicates that all baseline factors described in the first row of the table are included in the subsequent models.
